# Identification and Characterisation of the RalA-ERp57 Interaction: Evidence for GDI Activity of ERp57

**DOI:** 10.1371/journal.pone.0050879

**Published:** 2012-11-30

**Authors:** Adam Brymora, Iain G. Duggin, Leise A. Berven, Ellen M. van Dam, Basil D. Roufogalis, Phillip J. Robinson

**Affiliations:** 1 Cell Signalling Unit, Children’s Medical Research Institute, The University of Sydney, Sydney, Australia; 2 Faculty of Pharmacy, The University of Sydney, Sydney, Australia; IISER-TVM, India

## Abstract

RalA is a membrane-associated small GTPase that regulates vesicle trafficking. Here we identify a specific interaction between RalA and ERp57, an oxidoreductase and signalling protein. ERp57 bound specifically to the GDP-bound form of RalA, but not the GTP-bound form, and inhibited the dissociation of GDP from RalA *in vitro*. These activities were inhibited by reducing agents, but no disulphide bonds were detected between RalA and ERp57. Mutation of all four of ERp57’s active site cysteine residues blocked sensitivity to reducing agents, suggesting that redox-dependent conformational changes in ERp57 affect binding to RalA. Mutations in the switch II region of the GTPase domain of RalA specifically reduced or abolished binding to ERp57, but did not block GTP-specific binding to known RalA effectors, the exocyst and RalBP1. Oxidative treatment of A431 cells with H_2_O_2_ inhibited cellular RalA activity, and the effect was exacerbated by expression of recombinant ERp57. The oxidative treatment significantly increased the amount of RalA localised to the cytosol. These findings suggest that ERp57 regulates RalA signalling by acting as a redox-sensitive guanine-nucleotide dissociation inhibitor (RalGDI).

## Introduction

RalA belongs to the Ras family of small GTPases [1]. These are generally membrane-associated signalling proteins that cycle between an active GTP-bound state, which binds signalling molecules called effectors to propagate signalling, and an inactive GDP-bound state that does not bind effectors [2]. The GTPase cycle is tightly controlled by three main types of regulatory proteins: Guanine-nucleotide exchange factors (GEFs) that promote the dissociation of bound GDP to allow replacement with GTP, GTPase activating proteins (GAPs) that generate the inactive GDP-bound form, and Guanine-nucleotide dissociation inhibitors (GDIs) that bind and stabilise the GDP-bound form and block membrane binding to prevent activity of the small GTPase [3], [4].

GDIs effectively extract small GTPases from their active location on the surface of membranes by binding to the GTPase domain and by enveloping the C-terminal lipid adduct of the small GTPase, in what is thought to be a two-step process [5]–[8]. A cytosolic GDI-GTPase complex may then relocate to another intracellular site before disassociating, possibly with the aid of a release factor [9]–[11], allowing reactivation of the small GTPase via GEF activity [6], [12]. Whereas many specific GEF and GAP proteins exist for small GTPases, GDIs have only been described in detail for the Rab and Rho small GTPase families [4].

RalA is found on a variety of cellular membranes [13]. The closely related paralog RalB has several common activities with RalA, but the two proteins differ somewhat in sub-cellular and tissue distribution and cellular function [14], [15]. Ral proteins are involved in receptor-mediated endocytosis, through a Ral effector protein, RalBP1, and through Ral-stimulated phospholipase D (PLD) activity [13], [16]. Ral also controls exocytosis via an effector complex called the exocyst [17]–[19]. The exocyst is an eight-protein assembly involved in tethering secretory vesicles to specific sites on the plasma membrane where exocytosis is to occur [20]. The central position of the Ral proteins in vesicle trafficking events makes them important contributors to many cellular activities including polarized exocytosis, filopodia formation, cell migration, cytokinesis, autophagy, neurite branching, synaptic vesicle regulation and mitochondrial fission [1], [13], [15], [21]. Activated Ral is also a mediator of tumorigenesis [16].

Studies of the signalling pathways upstream of Ral have almost exclusively concerned its activators. Several GEFs have been described and the most characterised is RalGDS, a Ras effector protein [22]. There have also been a number of Ras-independent RalGEFs described [23], [24], and Ral can also be activated by intracellular Ca^2+^ [13]. In contrast, inhibitory regulators of Ral signalling are poorly understood. The existence of possibly two RalGAP proteins has been reported, but neither has been identified completely [25], [26]. Here, we report the identification of the multifunctional protein, ERp57, as a redox-sensitive specific binding protein of RalA that shows the hallmarks of a RalGDI.

## Materials and Methods

### Ethics Statement

The isolation of organs from animals was done with approval from the Animal Care and Ethics Committee for the Children's Medical Research Institute and The Children's Hospital Westmead (approval number C116), Sydney, Australia.

### Antibodies and other Materials

**Figure 1:**
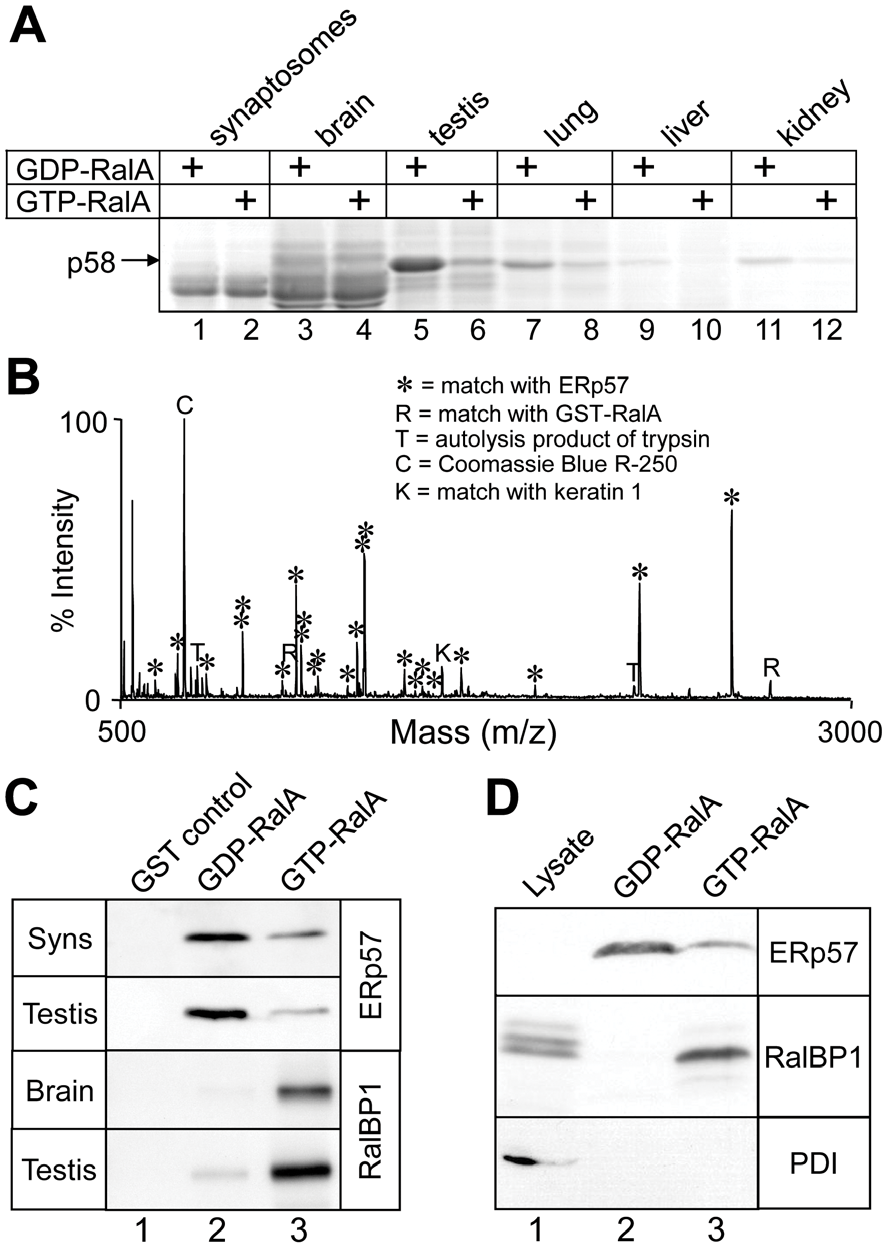
Isolation and identification of ERp57 as a GDP-RalA binding protein. (A) GST-RalA loaded with GDP or GTP was used as bait for pull-down experiments from the indicated rat tissue extracts. Bound proteins were analysed by SDS-PAGE and Coomassie blue staining. A GDP-dependent RalA binding protein, p58, is indicated. (B) MALDI-TOF mass spectrum of tryptic peptides derived from brain p58, showing peak identities, matched against theoretical digests with a mass accuracy of ±0.1 Da. (C) Western blotting of GST-RalA pull-down experiments from synaptosomes, whole brain or testis extracts, probed with anti-ERp57 or anti-RalBP1 antibodies, as indicated. (D) A whole testis lysate and a GST-RalA pull-down from testis extract were analysed by Western blot, probed with antibodies recognising ERp57, RalBP1 and PDI. Note that ERp57 was not clearly detected in the lysate in this experiment, due to the weak ERp57 antibody. Detection in pull-down assays was attributed to enrichment of ERp57 in these samples. In our subsequent work, we generated and used a stronger ERp57 antibody, which detects ERp57 in tissue extract (e.g., Fig. 3A).

Anti-RalA monoclonal and anti-RalB polyclonal antibodies were obtained from BD Transduction laboratories. Anti-6xHis tag polyclonal antibody was obtained from Santa Cruz Biotechnology, Inc. Anti-ERp57 polyclonal antibody was obtained from StressGen Biotechnologies (Figure 1), and for subsequent experiments rabbit anti-ERp57 sera were generated using peptide sequences from ERp57. Anti-GFP polyclonal and monoclonal antibodies were obtained from BD Biosciences Clontech. Polyclonal anti-PDI antiserum was a kind gift from Philip Hogg (University of New South Wales, Australia). All other antibodies were previously described [19]. *N*-acetylcysteine (NAC) and hydrogen peroxide solution were from Sigma Aldrich Pty Ltd.

### Recombinant RalA, ERp57 and Mutant Proteins

A fragment of human ERp57 cDNA, without the region encoding the N-terminal signal peptide (amino acids 1–23), cloned in a pET9 vector was provided by David Thomas (Montreal, Canada). pGEX-2T-RalA, containing simian RalA cDNA, was provided by Yoshito Kaziro (Yokohama, Japan). The RalA and ERp57 coding regions were subcloned into pDONR201 (Invitrogen Corp.) and subjected to site-directed mutagenesis when necessary (QuikChange, Stratagene). Clones were verified by DNA sequencing. The ORFs were further sub-cloned by recombination into pDEST15 (bacterial N-terminal GST-tag), pDEST17 (bacterial N-terminal 6xHis-tag) and pcDNA-DEST53 (mammalian N-terminal GFP-tag) expression vectors (Invitrogen). Expression of 6xHis-ERp57 in *Escherichia coli* and purification using Ni-NTA resin (Qiagen) were carried out according to the manufacturer’s protocol. Large quantities of plasmid DNA for transfection of mammalian cells were prepared using the Wizard Plus Maxipreps DNA purification system (Promega). GST-RalA beads were prepared as previously described [19]. When necessary, GST-RalA was eluted from GSH agarose with 50 mM Tris pH 8.0, 2.5 mM MgCl_2_, 20 mM reduced GSH, followed by dialysis into 20 mM Tris pH 7.4, 150 mM NaCl, 2.5 mM MgCl_2_.

### Pull-down Experiments, SDS-PAGE, Western Blotting, In-gel Digestion and MALDI-TOF MS

These were performed as described previously [19]. Densitometry was performed using ImageQuant *v*5.2 (Molecular Dynamics).

### Internal Edman Protein Sequencing

Bands of interest were excised from Coomassie Blue-stained polyacrylamide gels. Edman degradation of tryptic peptides was carried out by Newcastle Protein (University of Newcastle, Australia).

### GDI Activity Assays

The method was based on those previously described [27], [28]. For each condition, 4 µM GST-RalA (in 20 mM Tris pH 7.4, 150 mM NaCl, 2.5 mM MgCl_2_) was loaded with 1 µM [^3^H]-GDP in 10 µl of 20 mM Tris pH 7.4, 50 mM NaCl, 1% Triton X-100, 7.5 mM EDTA, 20 µg/ml leupeptin, 2 mM PMSF, 2x Complete protease inhibitor cocktail (Roche) at 37°C for 20 minutes. 1 M MgCl_2_ was then added to a final concentration of 10 mM, and the tubes were placed on ice for 5 minutes or until needed. The reaction was started by adding an equal volume of GDI assay buffer (20 mM Tris pH 7.4, 50 mM NaCl, 1% Triton X-100, 40 mM EDTA, 2 mM GDP) with or without recombinant 6xHis-ERp57. (The absence of Mg^2+^ allows the dissociation of nucleotides from a small GTPase at a reasonable rate.) The reaction was incubated for 10–60 minutes at 37°C. 20 µl aliquots were withdrawn from the bulk reaction at intervals, and then diluted in 500 µl of ice-cold washing buffer (20 mM Tris pH 7.4, 50 mM NaCl, 5 mM MgCl_2_) and stored on ice. Samples were filtered through a 0.2 µm nitrocellulose membrane under vacuum in an ice-cold manifold (Minifold II, Schleicher and Schuell). The membrane was washed three times with 0.5 ml of ice-cold washing buffer under vacuum and then removed and stored in a tray on ice for 3–5 minutes in ice-cold washing buffer. The membrane was blotted with filter paper, air dried, and then cut into individual samples which were incubated with 1 ml of 0.1 M HCl in scintillation vials for 20 minutes at room temperature. Scintillation fluid was added, the vials were shaken well, and then the samples were counted for tritium in a scintillation counter.

### Cell Culture and Transfections

A431 human epidermal carcinoma cells [29] were cultured in DMEM containing 10% foetal bovine serum (FBS). Cells were transfected using FuGENE 6 reagent according to the manufacturer’s instructions (Roche Molecular Biochemicals), using OPTI-MEM serum-free media (Gibco BRL). Cells were returned to the incubator for 5 hours. The cells were washed with phosphate buffered saline (PBS) and then incubated in DMEM medium with or without 20 or 30 mM NAC.

### Ral Activity Assay

The GST-RalBD plasmid construct (containing the Ral binding domain of RalBP1, amino acids 401–522) was obtained from Larry Feig (Boston, USA). GST-RalBD was overproduced in *E. coli* as described for GST-RalA [19]. A431 cells were seeded into 100 mm tissue culture dishes at ∼2×10^5^ cells/dish, and cultured to ∼75–80% confluency overnight. The cells were transfected, and if necessary, serum-starved overnight (as above) and the specified treatments were applied. The cells were washed twice with ice-cold PBS and then lysed on ice by the addition of ice-cold lysis buffer (25 mM Tris pH 7.4, 150 mM NaCl, 1% Triton X-100, 1 mM EGTA, 10 mM MgCl_2_, 20 µg/ml leupeptin, 1 mM PMSF). Cells were scraped and vortexed thoroughly. The lysates were centrifuged at 17,000 *g* for 10 minutes at 4°C. The supernatant was incubated with 10 µg GST-RalBD protein beads for 30 minutes at 4°C in micro-spin columns, with regular mixing, as previously described [30]. The lysate was collected by centrifugation and the beads were washed twice with lysis buffer (without protease inhibitors), then once with 20 mM Tris pH 7.4 containing 2.5 mM MgCl_2_. Protein was eluted with 30 µl SDS sample buffer, and analysed by SDS-PAGE and Western blotting using the indicated antibodies.

### Subcellular Fractionation

A431 cells were seeded into 100 mm tissue culture dishes at ∼2×10^5^ cells per dish, cultured to ∼75–80% confluency, and serum-starved (with or without NAC) overnight. The cells were stimulated with H_2_O_2_, and if necessary, washed twice with ice-cold PBS, and lysed on ice in 5 mM Tris pH 7.4 containing 5 mM MgCl_2_, 20 µg/ml leupeptin and 1 mM PMSF. The cells were scraped, and lysis was assisted by vigorous vortexing and passaging through a 30-gauge needle. The cell lysates were centrifuged at 100,000 rpm for 40 minutes at 4°C in a TLA120 bench-top ultracentrifuge rotor (Beckman). The supernatants (S100 cytosolic fractions) were removed and analysed by SDS-PAGE and Western blotting.

### Immunoprecipitation

For each reaction, a 10 µl bed volume of protein G-agarose beads (Roche) was coupled overnight to 1 µl anti-GFP polyclonal antiserum in 400 µl PBS with rotation at 4°C. A431 cell lysate was prepared as described for the Ral activity assays, and then a 20 µl aliquot was withdrawn for a Western blotting control. The lysates were then incubated with the anti-GFP antibody beads with mixing for 1 hour at 4 ^≫^C. The lysate was then removed by centrifugation using a Micro-spin column. The beads were washed three times with ice-cold lysis buffer (without protease inhibitors), and then twice with ice-cold 20 mM Tris pH 7.4, 2.5 mM MgCl_2_. 30 µl SDS sample buffer was then added, to elute bound proteins, which were subsequently analysed by SDS-PAGE and Western blotting.

## Results

### Identification of ERp57 as a RalA Binding Protein

To identify RalA binding proteins, we used a recombinant GST-RalA fusion protein coupled to glutathione (GSH) agarose in affinity chromatography (“pull-down”) experiments with various Triton X-100 solubilised rat tissue extracts. GST-RalA was loaded with either GTP or GDP immediately prior to use. With this approach we observed a ∼58 kDa protein that bound to GST-RalA in a GDP-dependent manner, which we initially referred to as p58 (Figure 1A). The amount of p58 that bound to RalA-GDP was greatest with rat testis extract, followed by lung, kidney, liver, brain and synaptosome extracts (Figure 1A). The p58 band from brain extracts was excised from the gel, and identified by matrix-assisted laser desorption ionisation time-of-flight mass spectrometry (MALDI-TOF MS, Figure 1B). The MALDI-TOF MS peptide mass data matched the hypothetical tryptic fragments of ERp57 (Genebank, gi|1352384) with a statistical probability of >99%. This identification was confirmed by MALDI-TOF MS post-source decay analysis of a peptide of *m/z*  = 1188.59 Da, which yielded a sequence of FV[Met-Ox]QEEFSR. This tryptic peptide corresponds to amino acids 335–343 of ERp57. We also identified the testis-derived p58 that bound to RalA, by both MALDI-TOF MS (data not shown) and Edman sequencing of four tryptic peptides. The peptides sequenced precisely matched the sequence of ERp57 at residues 141–152 (TEDEFKKFISDK), 259–270 (DLLTAYYDVDYE), 306–317 (TFSHELSDFGLE), and 352–362 (FLQEYFDGNLK). Western blotting confirmed that ERp57 bound specifically to RalA-GDP in both synaptosome and testis extracts, while the opposite and expected GTP-dependent binding was observed for the known RalA effector protein RalBP1 in a parallel experiment (Figure 1C).

ERp57 is a member of the Protein Disulfide Isomerise (PDI) family, which encompasses a highly versatile group of proteins that have an N-terminal signal sequence and at least one thioredoxin-like domain [31]. The best characterised member of this family is its namesake, PDI. PDI is the closest human paralog of ERp57, with 33% amino acid sequence identity and the same overall domain layout. Therefore, as a test of the specificity of the ERp57-RalA interaction, we examined GST-RalA pull-downs from rat testis extract for the presence of PDI. RalA bound ERp57 and RalBP1, as expected, but it did not bind PDI (Figure 1D, lanes 2 and 3), despite PDI’s presence in the lysate (lane 1). It is concluded that the binding of recombinant RalA-GDP by ERp57 in tissue extracts is specific, and is not a property of all PDIs.

### The ERp57-RalA Interaction is Direct and Redox-sensitive

**Figure 2:**
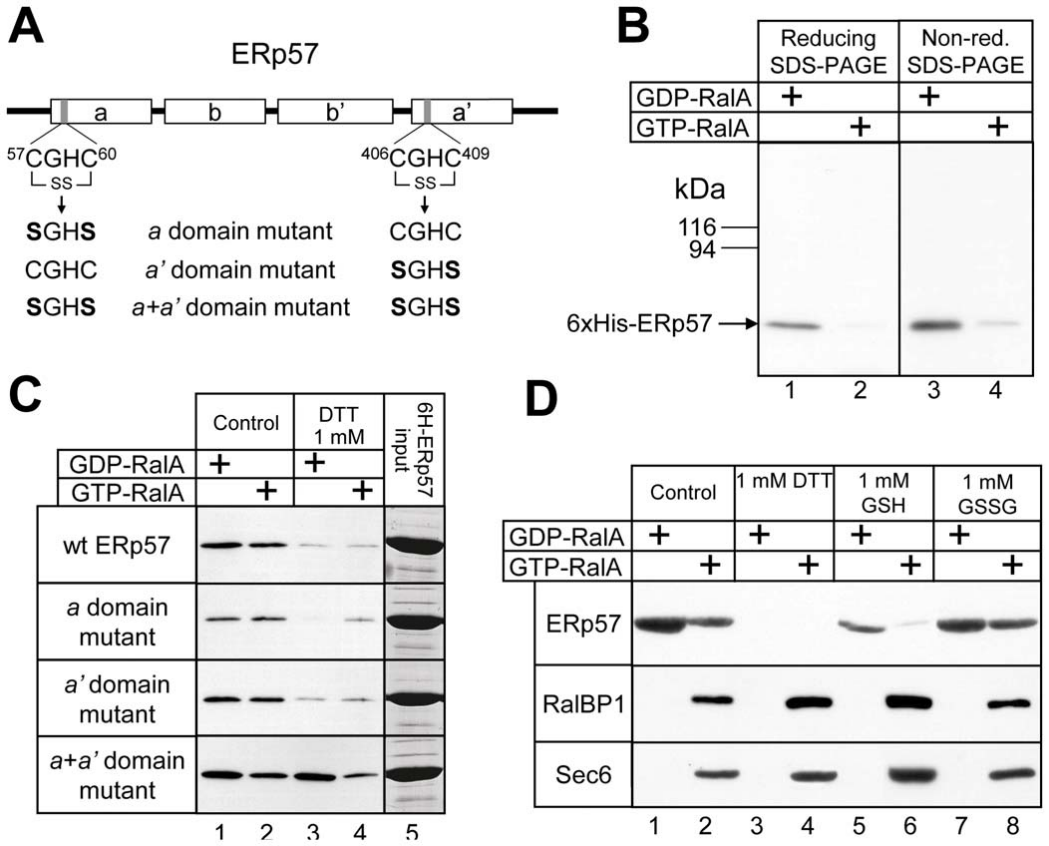
The ERp57-RalA interaction is direct and is redox-regulated. (A) The domain organisation of ERp57, showing the active cysteine thiol groups in their oxidised state (indicated by SS). The ERp57 catalytic site point mutations used are indicated. ERp57 also has a signal sequence at the N-terminus, and a nuclear localisation sequence (KPKKKKK) followed by an ER retrieval sequence (QEDL) at the C-terminus. (B) Purified 6xHis-ERp57 was used in GST-RalA pull-down experiments and was analysed by both reducing and non-reducing SDS-PAGE followed by Western blotting with an anti-6xHis-tag antibody. (C) 6xHis-ERp57 and mutants were purified and bound to GST-RalA loaded with GTP or GDP in the presence or absence of 1 mM DTT. The relative input levels of ERp57 or mutant proteins were analysed by Coommassie stained SDS polyacrylamide gels (lane 5). Results are representative of at least two independent experiments. (D) GST-RalA pull-down experiments using rat testis lysate in the presence of the indicated redox reagents were analysed by SDS-PAGE and Western blotting to detect ERp57, RalBP1 and Sec6.

We next tested whether the interaction between RalA and ERp57 was direct, or if it required the presence of some other factor from tissue lysates. A 6x-His tagged ERp57, lacking the N-terminal 23-amino-acid signal sequence (Figure 2A), was expressed and purified from *E. coli*, and then used in pull-down experiments with GST-RalA. Like tissue-derived ERp57, purified wild-type 6xHis-ERp57 bound RalA in a GDP-dependent manner (Figure 2B, lanes 1 and 2), demonstrating that their interaction is direct.

ERp57 has four thioredoxin-like domains, denoted (from the N- to the C-terminus) *a*, *b*, *b'* and *a'* (Figure 2A). The *a* and *a'* domains possess typical thioredoxin-like active site motifs (CGHC) that mediate disulfide exchange in specific substrate proteins, involving the two Cys residues. The *b* and *b'* domains lack this motif, but they retain the thioredoxin fold and are involved in substrate binding [32]. During its function as a protein disulfide isomerise, ERp57 forms an intermediate intermolecular disulfide bond with a substrate protein [33]. Although RalA does not contain any disulfide bonds that would require PDI activity for formation [34], we tested whether the ERp57-RalA interaction involved disulfide bonding. A GST-RalA pull-down experiment with purified 6xHis-ERp57 was analysed by non-reducing SDS-PAGE and Western blotting using antibodies recognising the 6xHis tag. No band was detected at a molecular weight of ∼100 kDa (the combined mass of 6xHis-ERp57 and GST-RalA) or greater (Figure 2B, lanes 3 and 4), suggesting that the ERp57-RalA interaction does not involve disulfide bonding between the two proteins.

ERp57 is a redox-sensitive protein, and cellular oxidative stress elicited with hydrogen peroxide induces oxidation of its active site thiols and *S*-glutathionylation [35], [36]. The oxidised state of ERp57 is required for its ability to bind DNA via the *a'* domain [37]. Similarly, we found that the binding of 6xHis-ERp57 to GST-RalA was strongly inhibited by the addition of the reducing agent dithiothreitol (DTT) (Figure 2C, top panel), suggesting that the oxidised form of ERp57 is required for strongest binding to RalA. Using this principle, we have developed a method for rapid purification of native ERp57 from rat testis lysate, by elution of bound ERp57 from GST-RalA-GDP affinity beads using DTT (data not shown).

To investigate the role of the *a* and *a'* thiol-containing domains of ERp57 in redox-state-dependent binding to RalA, we mutated the pair of active-site Cys residues in either one or both of the *a* and *a'* domains to a pair of Ser residues (Figure 2A). The binding to RalA was similar for 6xHis-ERp57 ("wt ERp57") and all three mutant proteins (Figure 2C, lane 1). Therefore, the cysteine residues are not essential for binding, despite the redox-state-dependence of the ERp57-RalA interaction. DTT-mediated inhibition of ERp57 binding to RalA was not greatly affected by individual mutation of either the *a* or the *a'* domain (Figure 2C, middle panels). However, the double domain mutant (*a*+*a'*), abolished the redox-sensitivity of the ERp57-RalA interaction (Figure 2C, lower panel). The above findings suggest that the conformation of ERp57 required to bind to RalA tightly is acquired in ERp57’s oxidised state, and this conformation is somehow mimicked by mutation of the pairs of active-site Cys residues to Ser. Since all four cysteine mutations are required to abolish DTT sensitivity, it would appear that the *a* and *a'* domains cooperate in some manner in binding to RalA.

To determine if the effect of reducing agents is specific to the ERp57-RalA interaction, we investigated their effect on the GTP-dependent binding of RalA to its effectors. A GST-RalA pull-down experiment using rat testis lysate was performed in the presence of various redox reagents and the RalA-interacting proteins were detected by Western blotting. Both DTT and the physiological reducing agent GSH inhibited the ERp57-RalA interaction, whereas the oxidised form of glutathione (GSSG) had no effect (Figure 2D). GTP-dependent binding to both RalBP1 and the exocyst complex (Sec6 antibody) were slightly elevated in the presence of either DTT or GSH, but were unaffected by GSSG (Figure 2D). This is probably due to a greater availability of RalA due to the absence of bound ERp57, but it is clear that no large changes in binding patterns were evident for RalBP1 and exocyst under the various redox conditions.

### The ERp57-RalA Interaction Involves the Switch II Region of RalA

Nucleotide-dependent conformational changes in RalA are essentially confined to two regions, switch I and switch II, that regulate interactions with RalA effectors [34], [38]. Switch II is frequently the primary binding site for the GDP-dependent binding of proteins to other small GTPases [5], [39], [40]. We therefore designed point mutations in RalA’s switch II region with the aim of identifying important amino-acid residues in RalA for ERp57 binding. The mutations D74A, Y75A, A77R and I78N were selected based on a modelled structure of RalA and a knowledge of previous mutations affecting the GDP-dependent interactions of other small GTPases [5], [41], [42]. The mutation D49A (from switch I) in RalA was included as a control that was previously reported to selectively lose interaction with RalBP1 and not the exocyst [18], [43].

**Figure 3:**
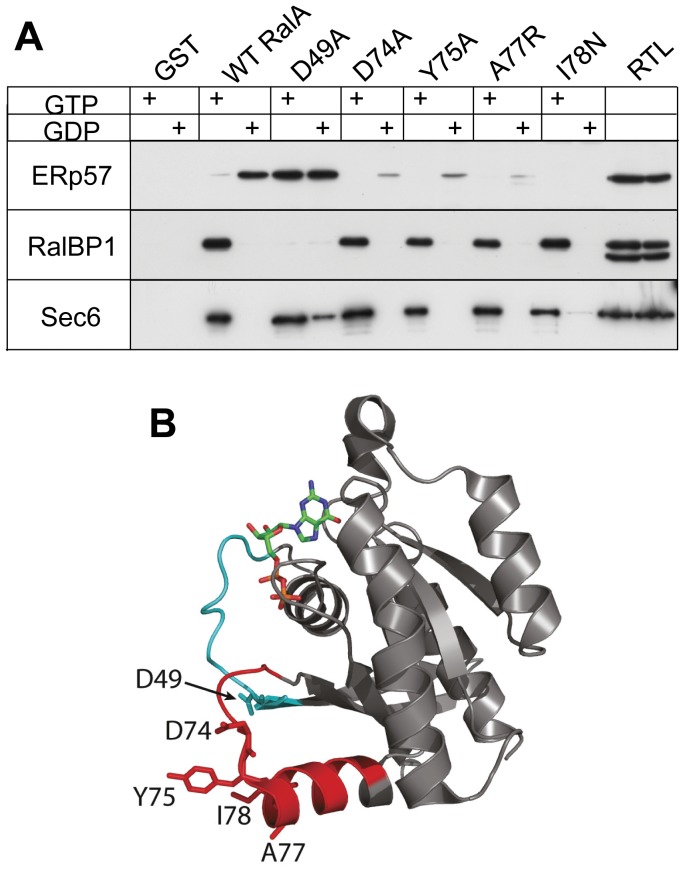
ERp57-RalA interaction involves the switch II region of RalA. (A) GST-RalA or the indicated mutants were loaded with GDP or GTP and then used as bait for pull-down experiments using rat testis lysate (RTL). Bound proteins were analysed Western blot using anti-ERp57, anti-RalBP1, or anti-Sec6 antibodies. The experiment shows three blots from the same pull-down experiment and results are representative of at least three independent experiments for each construct. (B) Crystal structure of GDP-bound RalA [55], indicating the switch I (cyan) and switch II (red) regions and the residues mutated for pull-down assays. (Pdb code: 1U90).

Purified GST-RalA and the various mutants were loaded with either GDP or GTP for use in pull-down experiments using rat testis extract. Interestingly, the RalA.D49A mutant efficiently bound ERp57, but the nucleotide dependence of ERp57 binding was completely lost (Figure 3A). Nucleotide-dependent binding by the exocyst (Sec6 antibody) was observed in the same sample (Figure 3A), whereas RalBP1 failed to bind both the GDP- and GTP-bound form of RalA.D49A, as expected [18], [43]. The behaviour of RalA.D49A strongly suggests that the regions of RalA that normally interact with ERp57 and RalBP1 are trapped in the GDP-bound conformation in this mutant, even in the presence of GTP. These findings confirm that some of the normal nucleotide-dependent conformational changes do not occur in RalA.D49A. Thus, the D49 residue is critical in transmitting the chemical information of the nucleotide to RalA’s structure and binding-partners. It is also clear that mutations of D49 would be expected to affect more than one Ral regulatory pathway.

The mutations in RalA’s switch II region (D74A, Y75A, A77R and I78N) greatly diminished binding to ERp57 (Figure 3A). Essentially complete loss of ERp57 binding was observed for I78N. In contrast, RalBP1 and the exocyst (Sec6) exhibited apparently normal GTP-dependent binding to all four switch II mutants (Figure 3A). We conclude that specific RalA-ERp57 binding involves the switch II region of RalA, and that switch I controls nucleotide-dependence of the RalA-ERp57 interaction.

### ERp57 Inhibits GDP Dissociation from RalA in a Redox-sensitive Manner

**Figure 4:**
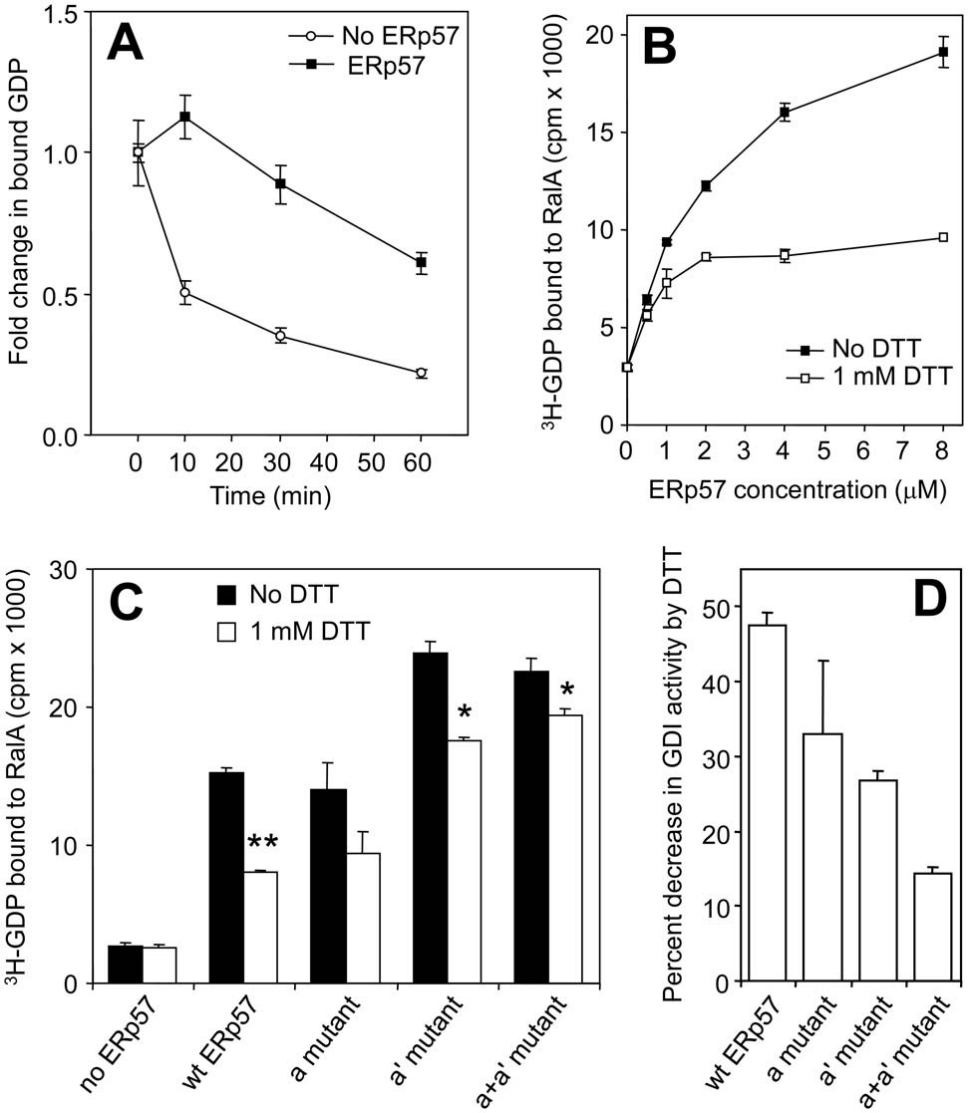
ERp57 has redox-sensitive RalGDI activity. (A) Time course of ^3^H-GDP dissociation from GST-RalA in the presence or absence of ERp57. 4 µM GST-RalA was pre-loaded with ^3^H-labelled GDP and incubated with or without 8 µM 6xHis-ERp57. Protein was collected by nitrocellulose filtration and the amount of bound ^3^H-GDP was determined by scintillation counting. Error bars represent SEM (*n* = 3). (B) GDI activity assays were performed as in (A) at the 30 minute reaction time, with and without the presence of 1 mM DTT at various ERp57 concentrations. Error bars represent SEM (*n* = 3). (C) GDI activity of 6xHis-ERp57 and mutants. GDI activity was determined with a 30 minute incubation time. Error bars represent SEM (*n* = 3), * represents p<0.05 and ** <0.01 using a two-tailed t-test relative to the no DTT sample in each case. (D) Percent decrease in GDI activity in the presence of DTT relative to the no-DTT experiment for each ERp57 variant, derived from the measurements in (C).

To determine if ERp57 is a regulator of Ral activity, we performed standard *in vitro* assays for GEF, GAP and GDI activities. We did not detect GEF or GAP activity of recombinant ERp57 (data not shown). However, GDI assays, based on monitoring the dissociation of radio-labelled GDP from RalA, revealed a redox-sensitive GDI activity of ERp57. Under control conditions (no ERp57), 21% of the initial RalA-bound ^3^H-GDP remained after 1 hour (Figure 4A). However, in the presence of 6xHis-ERp57, 63% of the original protein-bound ^3^H-GDP was retained after one hour (Figure 4A). We conclude that 6xHis-ERp57 inhibits the dissociation of GDP from RalA *in vitro*. We refer to this as RalGDI activity, however it is noteworthy that the usual definition of a GDI also includes a membrane removal activity (addressed further below).

Next, we examined the concentration dependence of the RalGDI activity of ERp57 in the presence and absence of DTT. The RalGDI activity of 6xHis-ERp57 was concentration dependent (Figure 4B, filled symbols). DTT inhibited RalGDI activity at all concentrations of 6xHis-ERp57, but the relative decrease was greatest at higher 6xHis-ERp57 concentrations (Figure 4B, open symbols). These data suggest that reduced ERp57 is a weak RalGDI compared to oxidised ERp57, consistent with the binding patterns described above.

To establish which of ERp57’s pairs of catalytic Cys residues might be responsible for the redox-sensitivity of RalGDI activity, the mutant ERp57 proteins (Figure 2A) were used in RalGDI assays. Both 6xHis-ERp57 and the *a* domain mutant inhibited GDP dissociation to a similar extent (Figure 4C). The GDI activity of the *a'* and the *a*+*a'* domain mutants were greater, suggesting that they might have a slightly higher affinity than wild-type ERp57. DTT had the effect of increasing GDP dissociation from RalA in all cases (Figure 4C), but the percent increase differed between the four proteins, as seen in the data summary shown in Figure 4D. Mutation of either the *a* or the *a'* domain Cys residues partly abolished the DTT-sensitivity of the GDI activity, compared to wild-type ERp57, but the affect of DTT on the *a*+*a'* domain mutant was relatively minor. It therefore appears that the oxidised state of the pairs of Cys residues in either domain individually contributes to RalGDI activity. This is consistent with the mutation binding studies (Figure 2C), where the *a*+*a'* domain mutant ERp57 was insensitive to the action of DTT to inhibit the ERp57-RalA interaction. These results suggest a hypothesis whereby oxidised ERp57 in a cellular context would display maximal RalGDI activity.

### Oxidative Treatment Inhibits Cellular RalA Activity via ERp57

Since oxidation of ERp57’s catalytic Cys residues is associated with maximal RalGDI activity *in vitro* (Figure 4), and ERp57 can be oxidised in cells following oxidative stress [35], the next series of experiments were designed to determine whether RalA activity is redox-sensitive in cells, and whether ERp57 is involved. We investigated RalA activity in A431 cells treated with hydrogen peroxide (H_2_O_2_), since A431 cells have characterised redox-signalling processes [44], [45]. We used an assay for measuring Ral activity that utilises the GST-tagged Ral binding domain (GST-RalBD) of RalBP1 to selectively isolate and measure the level of GTP-bound active RalA from cell lysates [46].

**Figure 5:**
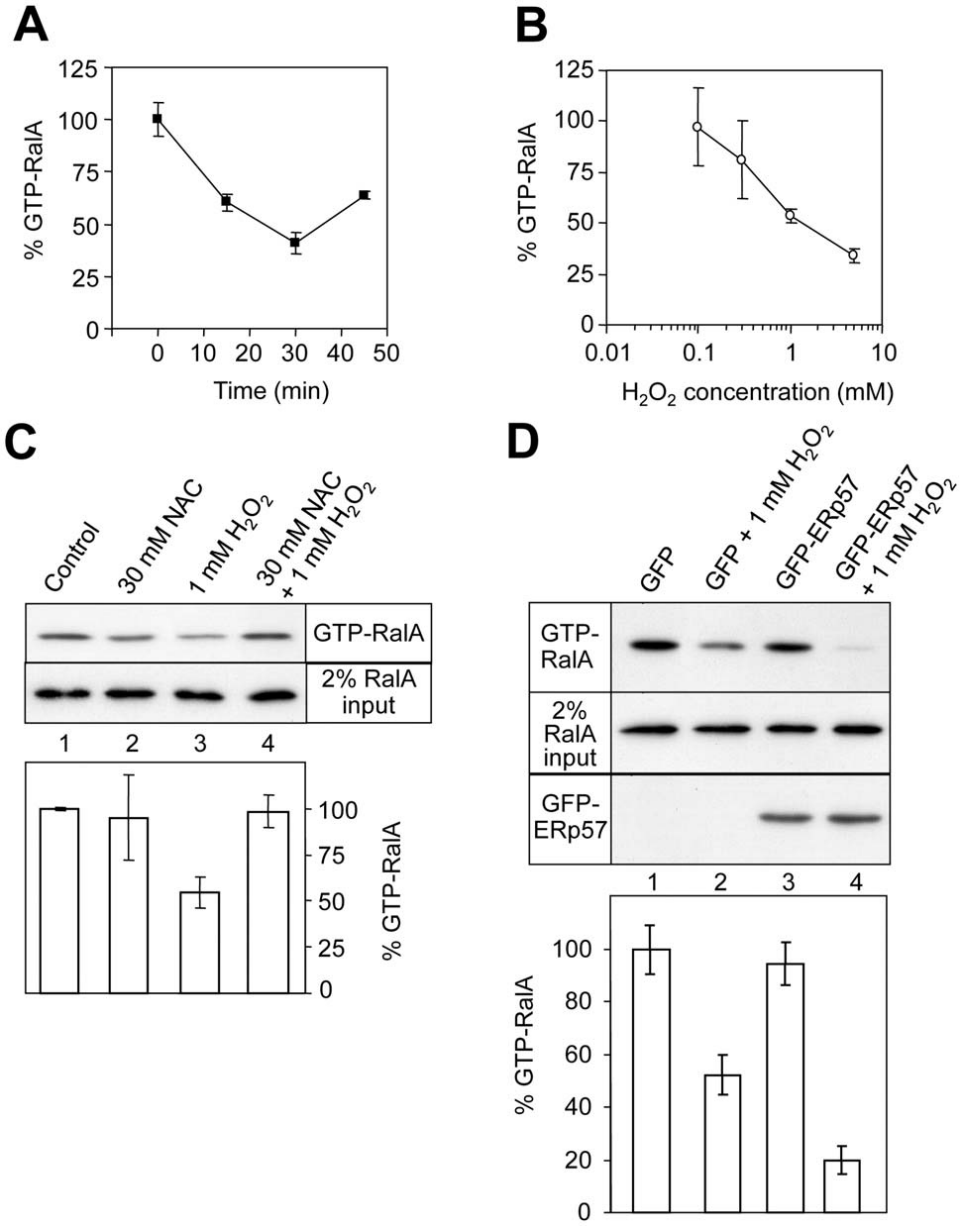
Oxidative stress inhibits cellular RalA activity via ERp57. GST-RalBD Ral activity assays monitoring the level of GTP-bound endogenous RalA (A) A431 cells were treated with 2 mM H_2_O_2_ and then the amount of active RalA-GTP was measured over 45 min. Error bars show SEM (*n* = 3). (B) Serum starved A431 cells were treated with various concentrations of H_2_O_2_ for 30 minutes and then RalA-GTP was measured (*n* = 3). (C) RalA activity assays examining the effect of pre-treatment with the antioxidant *N*-acetylcysteine (NAC) for 18 hours. The intensity of RalA staining in the upper panel indicates the level of RalA-GTP in a representative experiment, and the quantification of these bands by densitometry is shown beneath (*n* = 6). The relative levels of total cellular RalA are shown in the lower panel (from 2% of total protein loaded onto the GST-RalBD column). (D) Expression of GFP-ERp57 enhances the H_2_O_2_-induced inactivation of RalA in A431 cells. In the lower panel, error bars represent SEM (*n* = 9).

Recombinant GST-RalBD was coupled to GSH-agarose and used in pull-down experiments followed by Western blotting using an anti-RalA antibody. H_2_O_2_ treatment of serum-starved A431 cells decreased the basal level of RalA activity in a time and concentration dependent manner (Figures 5A, 5B). An inhibition of 50% of RalA activity was obtained by treatment of the cells with 1 mM H_2_O_2_ and maximal inhibition was reached after 30 minutes. Protection from oxidative stress can be achieved by the use of the cell-permeable antioxidant *N*-acetylcysteine (NAC) [44]. A431 cells appeared normal after overnight pre-treatment with 30 mM NAC, and this treatment did not significantly influence the basal level of RalA activation (Figure 5C, lanes 1 and 2). However, it abolished H_2_O_2_-induced RalA inactivation (lanes 3 and 4). None of the treatments altered the total level of cellular RalA (Figure 5C, lower panel).

To address whether the H_2_O_2_-induced RalA inhibition is mediated by ERp57, ERp57 (N-terminally GFP-tagged) was expressed in A431 cells that were then serum-starved and treated with 1 mM H_2_O_2_ for 30 minutes. In cells transfected with the GFP vector (pDEST53) alone, H_2_O_2_ decreased RalA activity as we saw previously (Figure 5D, upper panel, lanes 1 and 2). Transfection with GFP-ERp57 had no effect on basal RalA activity, but treatment of these cells with H_2_O_2_ elicited a much greater decrease in RalA activity than the GFP vector alone (compare lanes 2 and 4, Figure 5D). No change was observed in the total cellular level of RalA. Given that the transfection efficiency was routinely around 20–30% (data not shown), the expression of GFP-ERp57 greatly sensitised the transfected cells to the effect of H_2_O_2_ on RalA activity, but has little effect on RalA activity in the absence of the oxidative treatment. The results suggest that ERp57 participates in cellular inactivation of RalA in response to H_2_O_2_.

### Oxidative Treatment Affects the Subcellular Distribution of RalA

GDI proteins usually regulate the subcellular localisation of their target small GTPases by moving them from the membrane surface into the cytosol. This sequesters and maintains the inactive form of the small GTPase [7], [12]. ERp57 has a well-defined function in the maturation of certain glycoproteins in the endoplasmic reticulum (ER) and in redox-regulated interaction with tapasin during antigen presentation for the immune system [47], [48]. However, it is now clearly established that ERp57 has several additional roles in other regions of the cell, including the cytosol and nucleus [49]–[51], as well as the cell surface [52], [53].

**Figure 6:**
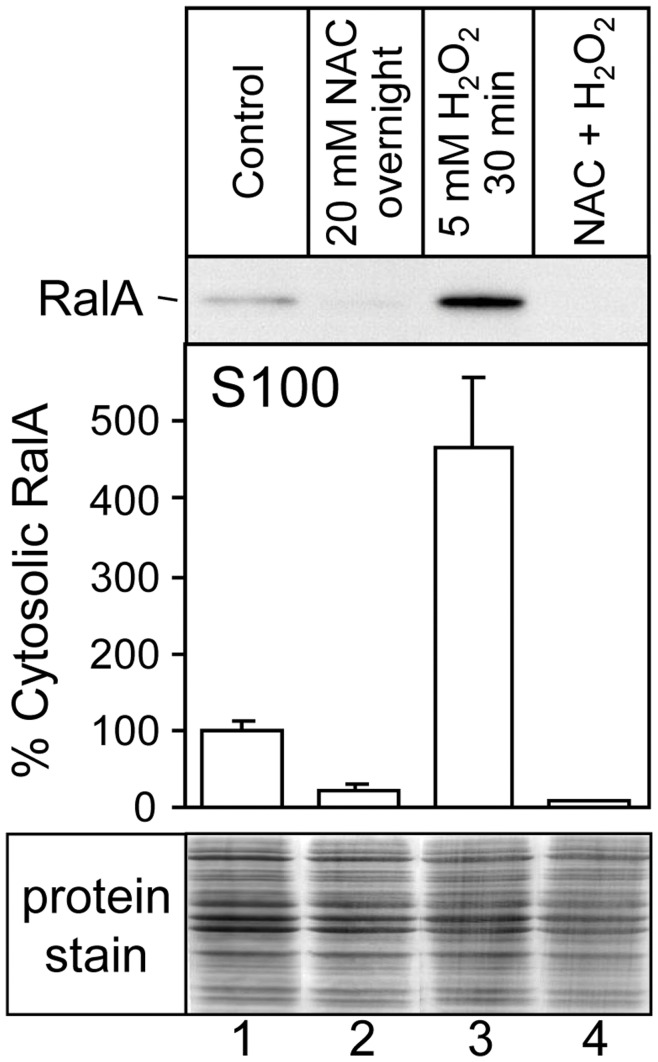
Redox reagents alter the subcellular distribution of RalA. A431 cells were treated with 5 mM H_2_O_2_ for 30 minutes, 20 mM NAC for 18 hours, or sequential treatment with 20 mM NAC for 18 hours and then 5 mM H_2_O_2_ for 30 minutes. Cell lysates were then fractionated by ultracentrifugation. The cytosolic fraction (S100) was analysed by Western blotting for the presence of endogenous RalA (upper panel), and quantitated by densitometry (central panel). Error bars represent the SEM (*n* = 3). The lower panel shows total cell protein stain of each of the samples represented in the upper panel.

To begin investigating the cellular localisation of RalA-ERp57 interactions, we tested the effects of oxidative treatment on RalA sub-cellular distribution. A431 cells were treated with various redox reagents, subjected to hypotonic lysis and separated into membrane and S100 cytosol fractions by ultracentrifugation (Figure 6). The S100 fractions were then analysed by SDS-PAGE and Western blotting for RalA. The basal level of cytosolic RalA (Figure 6, lane 1) represented approximately 1% of total cellular RalA (data not shown). Overnight pre-treatment of the cells with NAC reduced the level of cytosolic RalA to an even lower level (Figure 6, lane 2). In contrast, treatment of A431 cells with H_2_O_2_ led to a significant increase in the relative amount of cytosolic RalA (∼4.5 fold; Figure 6, lanes 1 and 3). NAC blocked the H_2_O_2_-mediated increase of cytosolic RalA (Figure 6, lane 4). The corresponding membrane fractions were also analysed for RalA, but no significant changes were observed (data not shown), reflecting the dominance of the membrane fraction in total RalA levels. These results indicate that a relatively small fraction of total cellular RalA is withdrawn from the membranes into the cytosol through oxidative treatment. This may occur in the case of a specifically localised signalling process.

## Discussion

We have discovered that ERp57 specifically interacts with the GDP-bound form of the small GTPase RalA. Despite its established role in the endoplasmic reticulum, ERp57 plays numerous signalling and other roles outside the endoplasmic reticulum [49]–[51], and it is considered most likely that RalA-ERp57 interactions would also take place outside the ER *in vivo*. We showed that ERp57 inhibited dissociation of GDP from RalA. Both RalA binding and RalA-GDP stabilisation by ERp57 were redox-sensitive *in vitro*, and this can be attributed to conformational changes in ERp57 in response to its redox environment, because mutation of the ERp57 active site cysteine residues to serine abolished redox-sensitivity of the ERp57-RalA interaction without the involvement of inter-molecular disulphide bonding between RalA and ERp57.

We mapped important residues of the RalA-ERp57 binding site to the switch II region of the GTPase domain of RalA, which in other small GTP binding proteins is involved in interactions with GDP-dependent binding partners such as GDIs [5], [41], [54]. In particular, the RalA.I78N mutant failed to bind ERp57 but still bound RalBP1 and the exocyst in the expected GTP-dependent manner. I78 is a specific tree-determinant residue for Ral within the Ras family [55], which are conserved residues within a given subfamily that differ from the other subfamilies, suggesting that ERp57 binding and GDI activity might be confined to the Ral sub-family.

Interestingly, the C3 exoenzyme from *Clostridium botulinum* (C3bot) binds with high specificity and affinity to RalA through the switch II region of RalA, and this causes a GDI-like effect similar to that observed here [56]. The structure of the RalA-GDP-C3bot complex demonstrated the direct involvement of at least two RalA switch II residues (D74 and Y75) at the protein-protein interface that were identified in the present study to be involved in the RalA-ERp57 interaction (Figure 4). This raises the possibility that the RalA-ERp57 interaction could be a target of the C3bot toxin.

ERp57 shows similar functionality to known GDI proteins. For example, RhoGDI interactions with the small GTPases Cdc42 and Rac1 also map to switch II [5], [57]. Furthermore, RhoGDIs require two domains for maximal GDI activity; likewise, ERp57 appears to require both the *a* and *a'* domain active-site cysteines for normal binding and activity, implying multiple interaction sites and raising the possibility of a stepwise binding and regulatory activity. Indeed the data in Figure 4 are consistent with the possibility that one domain of ERp57 may bind RalA to achieve the low GDI activity of reduced ERp57, followed by binding of the second domain in a redox-sensitive manner, to achieve maximal GDI activity.

ERp57 action on RalA shares the general feature of redox-sensitivity with RabGDIs. RabGDI-1 and -2 responded to oxidative treatment of adipocytes by dissociation from membranes, which is a key step in the regulatory activity of GDIs [58]. Conversely, antioxidant treatments lead to membrane retention of RabGDI, and the arrest of normal vesicle trafficking events within the cell [58]. Despite these functional parallels, ERp57 bears no clear homology with any of the known GDI proteins, and no homology exists between RabGDIs and RhoGDIs either [5], [57], highlighting the diversity seen in GDIs.

Recently, it was shown that a GDI-like activity of PDEδ is an important contributor to Ras sub-cellular distribution between membranes and cytosol; PDEδ expression increased Ras plasma membrane localisation, activity, and diffusion through the cytosol [59]. The ERp57-RalA system would appear to differ, as we found that ERp57 expression decreased the activity of RalA under oxidative conditions that also increased the levels of cytosolic RalA (Figures 5 and 6). Molecular characterisation of the ERp57-RalA interaction (Figures 2 and 3) also showed that there are significant differences in the binding specificity and mechanism between these systems [11].

Our investigation of endogenous cellular RalA found that it was sensitive to the effects of redox agents, in both its signalling activity and subcellular localisation (Figures 5 and 6). The regulation of cell function by redox-dependent signalling pathways has been well documented [60]. Ligand stimulation of many types of receptors results in the production of various reactive oxygen species (ROS) as intracellular second messenger species [61]. The production of ROS (such as H_2_O_2_) is constrained spatially, rather than influencing the overall redox state of the cell [60]. The experimental use of redox agents in cell culture broadly affects redox signalling pathways that physiologically only occur in distinct subcellular compartments or redox micro-environments [45]. Our experiments involving cellular oxidative treatment (H_2_O_2_) showed that supplementary expression of ERp57 significantly exacerbated the inactivation of RalA observed in response to H_2_O_2_. Therefore, our data support the notion that a localized oxidative signalling process oxidises ERp57 to result in a strong RalA-ERp57 interaction and GDI activity.
